# Rejecting conspiracies doesn't necessarily mean that you're smart: The relationship between conspiracy discrimination and reasoning ability

**DOI:** 10.1111/bjso.70117

**Published:** 2026-08-23

**Authors:** Samuel G. Robson, Kristy A. Martire, Samuel Pearson, Tylor J. Cosgrove, Jolanda Jetten, Kate Faasse, Matthew J. Hornsey

**Affiliations:** ^1^ School of Psychology University of New South Wales Sydney New South Wales Australia; ^2^ School of Psychology University of Queensland Brisbane Queensland Australia; ^3^ School of Business University of Queensland Brisbane Queensland Australia; ^4^ School of Psychology Adelaide University Adelaide South Australia Australia

**Keywords:** cognitive ability, cognitive sophistication, conspiracy belief, discriminability, reasoning, thinking style

## Abstract

Research examining the psychological predictors of conspiracy belief has overwhelmingly examined conspiracies that are unverified and/or implausible. Doing so potentially conflates ‘rational’ thinking with the tendency to heuristically reject conspiracist explanations. In three studies, we take a different approach, examining the predictors of the accuracy with which people can discriminate between unverified conspiracy theories and verified conspiracies (i.e. conspiracies that form part of the official record). The ability to discriminate between unverified and verified conspiracies was consistently higher among those with greater cognitive sophistication, as well as among political liberals, those lower in conspiracy mentality, and those with higher trust in experts, whereas associations with demographic variables were mixed. Conspiracy discrimination was also a much stronger predictor of cognitive sophistication than heuristic acceptance of conspiracy claims (response bias). In fact, good discriminators outperformed those who heuristically reject conspiracies (‘Indiscriminate Sceptics’) and those who heuristically endorse conspiracies (‘Indiscriminate Believers’) on each measure of cognitive sophistication. These findings challenge the idea that rejecting conspiracies is necessarily a sign of superior reasoning.

## INTRODUCTION

A central question in the psychology of conspiracy beliefs is why some people endorse them while others do not. Many conspiracy claims—for example, ‘global warming is a hoax’, or ‘the harmfulness of vaccines is being covered up’—run counter to evidence, and widespread belief in unsubstantiated conspiracies can have serious negative consequences for individuals and society (see Jolley et al., 2022). A widely supported finding in the literature is that conspiracy beliefs are associated with deficits in cognitive sophistication, including critical thinking, cognitive flexibility, scientific reasoning and scientific literacy (Binnendyk & Pennycook, 2022; Fasce & Picó, 2019; Georgiou et al., 2021; Gjoneska, 2021; Hornsey et al., 2023; Lantian et al., 2021; Pytlik et al., 2020; Yelbuz et al., 2022). This supports the idea that lower cognitive sophistication leads people to endorse conspiracy beliefs, and carries the implicit assumption that higher cognitive sophistication leads people to reject them.

One major challenge, however, is that definitions of ‘conspiracy theory’ are often imprecise and inconsistent across studies. Neutral or non‐normative definitions characterize conspiracy theories as explanations where the ultimate causes of important social and political events and circumstances are attributed to two or more powerful actors operating in secret (e.g. Douglas et al., 2019; Keeley, 1999). Such definitions are agnostic about whether a conspiracy theory is warranted or unwarranted, and therefore includes claims backed by little evidence (e.g. the Apollo moon landings were faked) as well as conspiracies that genuinely happened. The Watergate scandal, MK Ultra program and Iran–Contra affair are some examples of verified conspiracies (i.e. where protagonists have confessed, and the conspiracy is now part of the official historical record). Tobacco companies and the fossil fuel industry have also intentionally suppressed research conflicting with their interests (McGreal, 2021). Conspiracy thinking may even be adaptive, helping people detect dangerous outgroup coalitions (van Prooijen & van Vugt, 2018) or make sense of real scandals and abuses of power (Butter & Knight, 2020). Indeed, belief in conspiracies is higher in more corrupt countries, where conspiracies seem more plausible (Alper, 2023; Hornsey & Pearson, 2022).

In public discourse, however, conspiracy theories are typically perceived and stigmatized as implausible or bizarre claims that run counter to evidence (Douglas et al., 2021; Imhoff & Bertlich, 2024; Nera et al., 2020, 2022; Uscinski & Enders, 2023). Many academic definitions also build in epistemic value judgements. For instance, conspiracy theories have been defined as implausible (Brotherton, 2013), epistemically unwarranted (Lobato et al., 2014), lacking sufficient proof (Wagner‐Egger et al., 2019, 2023) and epistemically risky or ‘inherently prone to being false’ (Douglas & Sutton, 2023 p. 284). Psychological research has focused almost exclusively on studying belief in conspiracy theories as normatively defined (Imhoff & Bertlich, 2024), but this has several practical and epistemic problems (see Imhoff & Lamberty, 2020; Nera & Schöpfer, 2023). For one, if conspiracy theories are defined as unwarranted, endorsing them becomes functionally equivalent to endorsing claims that lack evidence. However, conspiracy theories defined in this way make up only a subset of all conspiracy claims, and a narrow focus risks conflating unwarranted conspiracy belief with conspiracy belief more broadly (e.g. Binnendyk & Pennycook, 2022; Brashier, 2023; Lantian et al., 2021).

Because prior work focuses almost exclusively on measuring belief in unwarranted conspiracy claims, the negative association between cognitive sophistication and conspiracy belief becomes somewhat circular, revealing only that less cognitively sophisticated people are more likely to believe implausible claims (Imhoff & Bertlich, 2024; Stanovich & Toplak, 2025). The implicit assumption is that rejecting conspiracies must therefore signal cognitive sophistication. If only belief in unwarranted conspiracy theories is assessed, however, it is unclear whether people disbelieve conspiracies because of the evidential basis of the claims or a heuristic tendency towards outright rejecting cospiracies. If cognitive sophistication plays a role, it should be reflected not in blanket rejection but in the capacity to discriminate well‐supported claims from those that lack evidence (see Stanovich & Toplak, 2025).

In a recent study, Frenken et al. (2024) presented participants with plausible and implausible conspiracy theories about a fictional country. Less reflective people were more likely to endorse implausible theories but endorsed plausible ones at rates comparable to their more reflective counterparts. In a belief‐updating task, those high in conspiracy mentality also continued to find conspiracy theories plausible despite strong contradictory evidence. Similarly, Hattersley et al. (2022) show that flawed reasoning is related to belief in implausible conspiracy theories but unrelated to plausible ones.

Examining plausible and implausible claims separately, however, provides an incomplete picture. This is because belief in plausible and implausible claims is often correlated (Bensley et al., 2020; Frenken et al., 2024; Moulding et al., 2016; Pennycook et al., 2025). Moreover, those higher in conspiracy mentality are more likely to endorse both (Frenken et al., 2024), reflecting general response tendencies rather than careful evaluation of each claim. Recent work has nevertheless begun to disentangle response tendencies from discernment. Tagand et al. (2026), for example, demonstrate that those low in conspiracy mentality more accurately distinguish warranted from unwarranted conspiracy claims, but also tend to reject epistemically warranted claims (see also Stanovich & Toplak, 2025).

In the present studies, we extend this work by examining how well people distinguish between verified and unverified conspiracies, and what this reveals about the relationship between conspiracy beliefs and cognitive sophistication. We define verified conspiracies as conspiracy claims supported by documented evidence and now form part of the official public record, whereas unverified conspiracies are conspiracy claims that lack compelling, supporting evidence. We define cognitive sophistication broadly as the capacity for complex thought, encompassing a constellation of cognitive abilities and thinking dispositions (see Toplak et al., 2014). Here we operationalize cognitive sophistication as general knowledge (breadth of factual knowledge about the world), cognitive reflection (the tendency to override incorrect intuitive responses through deliberation) and scientific reasoning (the ability to think critically about scientific evidence).

Our aim is to examine the relationship between cognitive sophistication and conspiracy belief in a way that disentangles the ‘accuracy’ (discrimination) of the beliefs from response bias (the tendency to endorse or reject conspiracy claims). We do this by examining the correlates and predictors of conspiracy discrimination and whether discrimination is a stronger predictor of cognitive sophistication than response bias. We then build on this in Studies 2 and 3 by assessing whether indiscriminate rejection of conspiracy claims, like indiscriminate belief, is associated with lower cognitive sophistication.

### Transparency and openness

Study 1 is an exploratory re‐analysis of unpublished data. Its analyses were not preregistered. Methods, hypotheses and planned analyses for Study 2 were preregistered prior to data collection (see https://osf.io/j9y4e/wiki?wiki=w3bgq) but the primary focus of Study 2 deviated from these original plans (details below). Materials, data, and analytic scripts for Studies 1 and 2 are available at: https://osf.io/pnavw/. Methods, hypotheses and planned analyses for Study 3 were preregistered prior to data collection (see https://osf.io/hdex4/wiki?wiki=6b82x), and all materials, data and analytic scripts can be found at: https://osf.io/xsjr2.

For each study, we report how we determined sample sizes, data exclusions and materials used. Data were analysed and visualized using R and R Studio. Each study received ethical approval from the UNSW Human Research Advisory Panel C. All participants provided informed consent. The authors declare no conflicts of interest.

## STUDY 1

### Methods

#### Design and procedure

This study used a correlational design. Participants first completed a general knowledge test before rating their belief in a range of unverified and verified conspiracies. They then completed an assessment of cognitive reflection and of scientific reasoning, before providing demographic information. The primary outcome variable was the degree to which participants discriminate between verified and unverified conspiracies. This was a re‐analysis of data from a study that primarily aimed to examine the effects of a reasoning checklist on how people evaluate a medical diagnosis. Half of the conspiracy items were presented before this reasoning task and half were presented after.

#### Participants

Participants were recruited via Prolific using a targeted recruitment strategy. Months prior to taking part, participants fluent in English (no geographical restrictions) completed a brief pre‐screen assessing belief in four implausible claims (from Martire et al., 2020; e.g. Global warming is a hoax). Those who rated any claim above 60 (out of 100) were invited to take part in this study as tentative ‘Endorsers’, whereas those who rated all claims below 40 were invited as tentative ‘Non‐endorsers.’ Recruitment was balanced across these groups to ensure strong representation of higher‐endorsing participants, who might be underrepresented in general population samples (see Robson, Faasse, Gordon, et al., 2024; Van Prooijen et al., 2023). In all, the sample included 247 participants after two were excluded for not passing at least two of three attention checks. Post‐hoc sensitivity analyses indicate that a sample of this size is sufficient to detect small‐to‐medium effects with 80% power (*r* = .18; *f* ^2^ = .06).

Participants were primarily from the United States (26.7%) and the United Kingdom (23.1%), but also from other countries (10.1% from South Africa, 6.9% from Poland, 5.7% from Portugal; 4.5% from Australia and 23.1% from elsewhere). The mean age of the participants was 36.5 (*SD* = 13.2). Most (73.3%) identified as male whereas 25.1% identified as female (1.2% other; 0.4% did not say). A majority of the sample (70.9%) identified as White/Caucasian, followed by African American (9.3%), Hispanic (6.9%) and Asian (5.7%; 5.7% other; 1.6% did not say). Most of the participants (69.2%) had tertiary level education or higher (29.6% secondary education or less; 1.2% did not say). Most of the sample (64.0%) also reported English as their first language (36.0% said it was not).

#### Materials

##### Conspiracy belief

Participants rated their belief in 32 conspiracy claims—16 unverified and 16 verified. Unverified items were adapted from the Belief in Conspiracy Theory Inventory (BCTI; Swami et al., 2011) and verified items were drawn from Pennycook et al. (2025), with one item added. Items were divided into two sets. Each set included eight unverified conspiracies, eight verified conspiracies and one attention check. Participants completed both sets in a counterbalanced order; one set was presented before a reasoning task, and the other after, and items were in a different random order for each participant. The reasoning task itself is beyond the scope of the study, but materials are available on the OSF. Mean scores were calculated separately for the unverified and verified items. Belief was rated on a sliding scale ranging from 0 (Completely false) to 9 (Completely true).

##### General knowledge

Eight general knowledge questions were presented in a randomized order. All responses were provided using a scale from 0 (Not at all true) to 100 (Definitely true). A general knowledge score was computed by first reverse coding false items and then computing the mean, out of 100, across all eight questions. An example item was ‘spiders have six legs’. One attention check was also included (drag the slide to Definitely true).

##### Scientific reasoning scale (SRS)

The SRS (Drummond & Fischhoff, 2017) consists of 11 true/false questions designed to assess individual differences in scientific reasoning ability. This is the capacity to evaluate scientific findings with respect to the factors that determine their quality (e.g. control groups, confounds, causal inference). Scores were computed by summing the number of correct answers (range: 0–11).

##### Cognitive reflection test (CRT)

Participants answered seven CRT questions (Frederick, 2005; Thomson & Oppenheimer, 2016). These are designed to assess the capacity to suppress an incorrect intuitive (‘system 1’) answer to a question in favour of a correct reflective (‘system 2’) answer. For example, ‘a bat and a ball cost $1.10 in total. The bat costs $1.00 more than the ball. How much does the ball cost?’ The incorrect intuitive answer is 10c, whereas the correct reflective answer is 5c. Scores were calculated by summing the number of correct answers (range: 0–7). Participants also answered follow‐up questions about their confidence and the strategies they used.

##### Demographics

Participants were asked to provide their age, gender, ethnicity, level of education, and whether English was their first language. Only the woman/female (0) and man/male (1) gender categories were included in analyses, and education was converted to a five‐point ordinal scale.

### Results and discussion

#### Conspiracy discrimination and response bias

The primary dependent variable was an index of discrimination between verified and unverified conspiracies based on Signal Detection Theory (SDT). SDT can be applied in this context because a participant says that a claim is either false (responses 0–4) or true (responses 5–9), and each conspiracy is either verified or unverified (a proxy for true and false in reality). This means there are four potential outcomes: a person says a verified conspiracy is true (Hit), a verified conspiracy is false (Miss), an unverified conspiracy is false (Correct rejection), or an unverified conspiracy is true (False alarm). Better discrimination means that a person tends to endorse verified conspiracies more than unverified ones.

Unlike many other signal detection metrics that dichotomize responses into correct/incorrect or yes/no (e.g. *d'*), we quantified discriminability via the empirical area under the curve (AUC). Unlike *d'*, AUC does not rest on assumptions about underlying distributions. Instead, it leverages the full range of the response scale. This is particularly well suited to our data because belief is measured via graded levels of confidence rather than binary judgements. For the AUC method, a curve is constructed by plotting the hit rate against the false alarm rate across all decision thresholds (points along the response scale). The area underneath the curve can then be estimated numerically. We used the *pROC* package in R (Robin et al., 2011) to estimate AUC, which sums the areas of a series of adjacent trapezoids based on points at each decision threshold. An AUC of 0.5 reflects chance‐level performance, whereas values approaching 1 indicate stronger discrimination.

As a secondary measure, we computed each participant's response bias. This is the tendency to say true versus false (calculated as *c*; Macmillan & Creelman, 2005). Values greater than zero reflect a liberal response bias (tend to say ‘true’) whereas values less than zero reflect a conservative response bias (tend to say ‘false’).

#### Relationships between variables

We first examined zero‐order correlations across the entire sample (Table 1). Unsurprisingly, belief in unverified conspiracies, belief in verified conspiracies, and response bias were strongly positively intercorrelated. Consistent with Frenken et al. (2024), cognitive measures like scientific reasoning and cognitive reflection were significantly associated with reduced belief in unverified conspiracy theories. However, belief in verified conspiracies was associated with higher general knowledge scores. Additionally, better discrimination between verified and unverified conspiracies (AUC) was associated with better scientific reasoning, cognitive reflection and general knowledge, and identifying as male.

**TABLE 1:** Descriptives statistics and zero‐order correlations between variables in Study 1.

	*M*	*SD*	1	2	3	4	5	6	7	8	9	10
1. Unverified consp. belief	3.54	2.18	–									
2. Verified consp. belief	4.47	1.94	0.69**	–								
3. Response bias (c)	−0.11	0.87	0.88**	0.86**	–							
4. Discriminability (AUC)	0.58	0.16	−0.55**	0.18**	−0.24**	–						
5. General knowledge	83.64	13.62	−0.09	0.14*	0.01	0.25**	–					
6. Scientific reasoning	6.31	2.34	−0.29**	−0.05	−0.19**	0.31**	0.20**	–				
7. Cognitive reflection	4.09	2.05	−0.22**	−0.03	−0.16*	0.25**	0.23**	0.38**	–			
8. Age	36.51	13.25	0.05	0.14*	0.15*	0.05	0.09	0.03	0.06	–		
9. Education	2.84	0.73	−0.07	−0.06	−0.05	0.01	0.00	0.03	0.03	0.10	–	
10. Gender	‐	‐	−0.10	0.05	−0.06	0.19**	−0.01	0.14*	0.23**	−0.14*	−0.03	–
11. Native English	‐	‐	0.17**	0.15*	0.19**	−0.07	−0.00	−0.02	−0.13*	0.42**	−0.04	−0.10

*Note*: For analyses of gender, 0 = woman/female, 1 = man/male. For analyses of English‐speaking status, 0 = non‐native speaker, 1 = native speaker.

* *p* < .05;

** *p* < .01.

#### Predicting discriminability

We formally examined the unique predictors of discriminability, controlling for other variables, using multiple linear regression analysis. The predictors together explained 18.0% of variance in discriminability. As illustrated in Table 2, scientific reasoning, general knowledge and identifying as male predicted better discrimination, consistent with the zero‐order correlations above. However, CRT performance was not a significant predictor; nor were age, education and English‐speaking status.

**TABLE 2:** Predicting the ability to discriminate between verified and unverified conspiracies (AUC) in Study 1.

Predictor	*β*	*b*	*SE*	*t*‐value	*p*‐value
Intercept	–	0.22	0.08	2.94	.004
General knowledge	.19	0.00	0.00	3.15	.002
Scientific reasoning	.24	0.02	0.00	3.66	<.001
Cognitive reflection	.08	0.01	0.01	1.14	.257
Age	.07	0.00	0.00	1.05	.295
Education	−.02	0.00	0.01	−0.29	.772
Gender	.14	0.05	0.02	2.18	.030
Native English	−.08	−0.03	0.02	−1.17	.243

*Note*: For analyses of gender, 0 = woman/female, 1 = man/male. For analyses of English‐speaking status, 0 = non‐native speaker, 1 = native speaker.

#### Predicting cognitive sophistication

We also flipped the analytic direction by conducting linear regression analyses to assess whether AUC more strongly predicts cognitive sophistication than response bias. Response bias alone did not significantly predict general knowledge (*b* = 0.21, *p* = .830), but it was a significant negative predictor for cognitive reflection (*b* = −0.38, *p* = .010) and scientific reasoning (*b* = −0.50, *p* = .003). When AUC was added as a second predictor, however, response bias no longer significantly predicted any cognitive measure (general knowledge: *b* = 1.23, *p* = .218; cognitive reflection: *b* = −0.25, *p* = .091; scientific reasoning: *b =* −0.32, *p* = .055). AUC, by contrast, was consistently a significant, positive predictor of cognitive sophistication (general knowledge: *b* = 22.64, *p* < .001; cognitive reflection: *b* = 2.87, *p* < .001; scientific reasoning: *b =* 4.09, *p* < .001).

Consistent with Frenken et al. (2024), cognitive variables like scientific reasoning and cognitive reflection ability predicted lower belief in unverified conspiracies. Unlike Frenken and colleagues, however, we integrated belief ratings into a measure of discriminability. General knowledge, scientific reasoning and cognitive reflection positively correlated with discriminability, as did gender (with men scoring higher than women). Flipping the analytic direction also revealed that response bias did not predict performance on cognitive measures above and beyond discriminability. Together, these results suggest that sophisticated reasoning is not simply about rejecting unverified conspiracies, but about differentiating claims with empirical support from those without. This has been obscured by the near exclusive focus on unverified or implausible conspiracy claims in past research.

## STUDY 2

In Study 1, we uncovered some cognitive and demographic predictors of conspiracy discrimination. In Study 2, we replicate and extend these findings with a larger sample. Moreover, to better understand what underlies conspiracy discrimination, we include additional predictors linked to conspiracy belief, including conspiracy mentality, trust in experts, political orientation and socio‐economic status (see Brotherton et al., 2013; Enders et al., 2024; Frenken et al., 2024; Imhoff et al., 2022; Van Prooijen et al., 2015; Vranic et al., 2022). We also re‐assess the relationship between cognitive sophistication and conspiracy beliefs using additional analyses.

Similar to Study 1, we disproportionately recruited people who endorse fringe conspiracies, preregistering several analyses comparing ‘Endorsers’ to ‘Non‐endorsers’. However, it became clear post‐data collection that comparing three groups is more appropriate: those who indiscriminately believe conspiracies, those who indiscriminately reject conspiracies (including verified ones), and those who accurately discriminate between the two. We therefore begin by replicating the Study 1 analyses, before comparing these three groups on cognitive sophistication, and then reporting the preregistered analyses.

### Methods

#### Design, materials and procedure

Study 2's methods were similar to Study 1. Participants were pre‐screened in the same way. In the study itself, participants first responded to 32 conspiracy items presented in random order, but this time there was no intervening reasoning task. Conspiracy items were taken from Study 1, but some were reworded to improve clarity or replaced to avoid overlap with the pre‐screen. Participants then completed a general knowledge test, which we expanded to 16 items (8 true, 8 false), followed by the CRT and SRS.

This was followed by the Conspiracy Mentality Questionnaire (CMQ), a five‐item measure designed to assess differences in the tendency to engage in conspiracist ideation (Bruder et al., 2013). Responses were made on an 11‐point scale ranging from 0 (Certainly not) to 100 (Certain). An example item is ‘I think that many very important things happen in the world, which the public is never informed about.’ Scores were calculated by averaging across all five items (range: 0–11).

Participants then completed a five‐item scale measuring trust in expert authority (Robson, Faasse, & Martire, 2024), which has good internal consistency (*α* = .86). Responses were made on a seven‐point scale from 1 (Strongly disagree) to 7 (Strongly agree). An example item is ‘I tend to follow what experts say even if I don't fully understand their reasoning.’ We computed a mean score across all five items (range: 1–7).

Finally, participants indicated their political orientation on social and economic issues (1 = Left/Liberal, 7 = Right/Conservative), which we averaged to form a combined measure of conservatism (*r* = .76). They then answered demographic questions, which were the same as in Study 1 but with the addition of the MacArthur Scale of Subjective Social Status (Adler et al., 2000), which assesses perceived socio‐economic status (SES; range: 1–10).

#### Participants

We preregistered that we would recruit at least 600 participants, but aimed for as many as possible, with the stopping rule that recruitment would cease by the end of May 2024. We ended up with a sample of 663 participants after six were excluded for failing at least two attention checks (as preregistered). Post‐hoc sensitivity analyses indicate that this sample size provides 80% power to detect effect sizes of *r* = .11 and *f* ^2^ = .026.

Participants were primarily from the United States (22.2%) and the United Kingdom (16.7%), but also from other countries (10.1% from South Africa, 9.2% from Canada, 7.4% from Portugal, 6.8% from Mexico, 5.9% from Australia, and 21.7% from elsewhere). The mean age of participants was 39.0 (*SD* = 14.1). Most (56.0%) identified as male (42.1% female; 1.8% other; 0.2% did not say). Most (65.9%) identified as White/Caucasian (5.0% African American, 8.9% Hispanic; 9.7% Asian; 9.4% Other; 1.2% did not say). A majority (76.0%) also had tertiary level education or more (23.4% secondary education or less; 0.6% did not say), and most (59.9%) reported English as their first language (40.1% said it was not).

### Results and discussion

#### Relationships between variables

Table 3 displays the zero‐order correlations. Like Study 1, there were strong positive intercorrelations between belief in unverified conspiracies, belief in verified conspiracies and response bias. Consistent with prior literature (Frenken et al., 2024), measures of cognitive ability (general knowledge, scientific reasoning and cognitive reflection) were associated with lower belief in unverified conspiracies, but unrelated to belief in verified conspiracies. In line with Study 1, distinguishing between verified and unverified conspiracies (AUC) was associated with higher scores on these cognitive measures. Better discriminators were also less conspiracy‐minded, more trusting of experts, more politically liberal and skewed male.

**TABLE 3:** Descriptive statistics and zero‐order correlations between variables in Study 2.

	*M*	*SD*	1	2	3	4	5	6	7	8	9	10	11	12	13	14
1. Unverified consp. belief	3.59	2.17	–													
2. Verified consp. belief	4.79	1.87	0.70**	–												
3. Response bias (c)	−0.05	0.87	0.90**	0.86**	–											
4. Discriminability (AUC)	0.61	0.16	−0.61**	0.08*	−0.34**	–										
5. General knowledge	73.23	13.36	−0.20**	0.06	−0.08*	0.32**	–									
6. Scientific reasoning	6.41	2.65	−0.23**	−0.03	−0.14**	0.29**	0.30**	–								
7. Cognitive reflection	4.05	1.99	−0.19**	0.01	−0.10*	0.28**	0.36**	0.43**	–							
8. Conspiracy mentality	6.68	10.67	.67**	0.49**	0.64**	−0.41**	−0.16**	−0.25**	−0.19**	–						
9. Trust in experts	4.40	1.47	−0.49**	−0.30**	−0.41**	0.37**	0.10*	0.09*	0.18**	−0.36**	–					
10. Conservatism	4.01	1.76	0.29**	0.10**	0.22**	−0.31**	−0.18**	−0.14**	−0.12**	0.28**	−0.48**	–				
11. SES	5.31	1.64	−0.04	−0.08*	−0.06	0.00	0.06	0.07	0.04	−0.07	0.06	0.00	–			
12. Age	39.02	14.08	0.05	0.12**	0.10**	0.02	−0.09*	0.06	−0.05	0.00	−0.28**	0.31**	−0.03	–		
13. Education	2.98	0.74	−0.02	−0.02	−0.01	0.02	0.05	0.05	−0.01	−0.06	0.11**	−0.07	0.21**	0.02	–	
14. Gender	–	–	−0.08*	0.02	−0.05	0.16**	0.06	0.06	0.19**	−0.07	0.10**	−0.03	−0.02	−0.15**	−0.05	–
15. Native English	–	–	0.10**	0.12**	0.13**	−0.03	−0.13**	0.02	−0.12**	0.11**	−0.24**	0.26**	−0.10*	0.45**	−0.07	−0.13**

*Note*: For analyses of gender, 0 = woman/female, 1 = man/male. For analyses of English‐speaking status, 0 = non‐native speaker, 1 = native speaker.

* *p* < .05;

** *p* < .01.

#### Predicting discriminability

We used linear regression analysis to explore unique predictors of conspiracy discriminability (see Table 4). The predictors together explained 34.6% of the variance in discriminability. Consistent with Study 1, general knowledge and scientific reasoning predicted better discrimination. Better discriminators also tended to be less conspiracy‐minded, more trusting of experts, more politically liberal and skewed male. In contrast to Study 1, age was a significant positive predictor of accurate discrimination.

**TABLE 4:** Predicting discrimination of verified and unverified conspiracies (AUC) in Study 2.

Predictor	*β*	*b*	*SE*	*t*‐value	*p*‐value
Intercept	–	0.39	0.06	6.74	<.001
General knowledge	.21	0.00	0.00	5.98	<.001
Scientific reasoning	.08	0.00	0.00	2.25	.025
Cognitive reflection	.06	0.01	0.00	1.69	.092
Age	.13	0.00	0.00	3.32	.001
Education	−.02	0.00	0.01	−0.53	.597
Gender	.11	0.04	0.01	3.33	.001
Native English	.07	0.02	0.01	1.95	.051
Conspiracy mentality	−.22	−0.02	0.00	−6.22	<.001
Trust in experts	.24	0.03	0.00	6.07	<.001
Political conservatism	−.13	−0.01	0.00	−3.38	.001
SES	−.03	0.00	0.00	−0.97	.331

*Note*: For analyses of gender, 0 = woman/female, 1 = man/male. For analyses of English‐speaking status, 0 = non‐native speaker, 1 = native speaker.

#### Predicting cognitive sophistication

Like Study 1, we flipped the analytic direction and conducted linear regression analyses to examine whether AUC is a stronger predictor of cognitive sophistication than response bias. We conducted regression analyses with only response bias as a predictor and compared them to regressions with both AUC and response bias as predictors. Response bias significantly negatively predicted general knowledge (*b* = −1.20, *p* = .044), scientific reasoning (*b* = −0.43, *p* < .001) and cognitive reflection (*b* = −0.23, *p* = .010). When AUC was added as a predictor in each instance, however, response bias was no longer significantly predictive (*p*s > .05), whereas AUC was consistently a significant, positive predictor (general knowledge: *b* = 28.13, *p* < .001; scientific reasoning: *b* = 4.62, *p* < .001; cognitive reflection: *b* = 3.46, *p* < .001).

#### Comparing correlations with conspiracy mentality

Because conspiracy mentality is a widely used predictor of conspiracy beliefs, we conducted a dependent samples Fisher's *r*‐to‐z test comparing conspiracy mentality's association with response bias and with discriminability. Conspiracy mentality was more strongly associated with response bias than with discriminability, *z* = 32.25, *p* < .001, suggesting that conspiracy mentality primarily captures a tendency to endorse conspiracy claims rather than reduced discriminability.

#### Comparison of three groups

Consistent with prior research, we found that those higher in cognitive sophistication are less likely to believe unverified conspiracies. However, this relationship in isolation risks oversimplifying the picture because cognitive sophistication was also associated with *discriminating between* verified and unverified conspiracies. Among those who disbelieve unverified conspiracies, there may be two kinds of people: those who accurately differentiate between real and false conspiracies, and those who reject conspiracies indiscriminately. It is therefore unclear whether the link is driven by heuristic rejection or effective discrimination.

To test this idea, we categorized participants into three groups based on their conspiracy discrimination (AUC) and response bias (c) scores. ‘Indiscriminate Believers’ were those with a liberal response bias (*c* > 0) and poor discrimination (AUC < .7). ‘Indiscriminate Sceptics’ were those with a conservative response bias (*c* ≤ 0) and poor discrimination (AUC < .7). ‘Good Discriminators’ were those with high discrimination scores (AUC ≥ .7). We used these threshold values because an AUC of 0.7 is considered ‘reasonable’ (Swets, 1988) and a response bias of zero is the unbiased midpoint.

**FIGURE 1:**
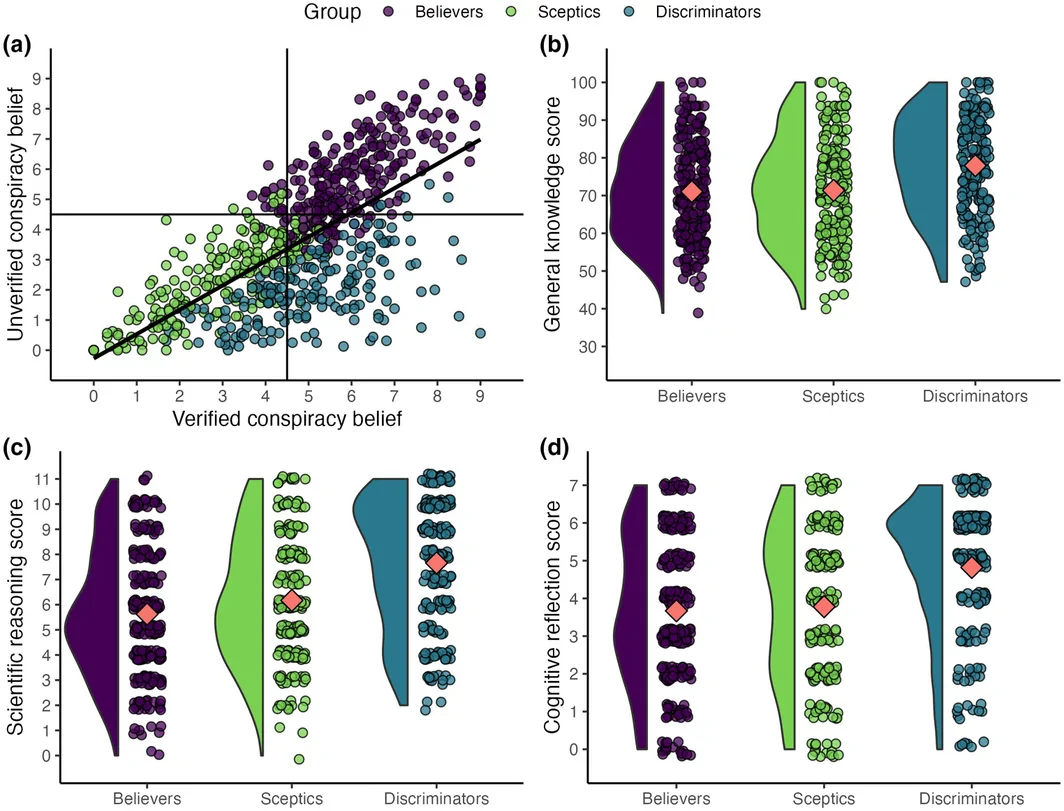
Comparison of three groups on cognitive ability measures in Study 2. In Panel a, the three groups—Indiscriminate Believers (purple), Indiscriminate Sceptics (green) and Good Discriminators (dark blue)—are plotted according to their mean belief in unverified and verified conspiracies. The regression line represents the correlation between the two variables. General knowledge (b), scientific reasoning (c), and cognitive reflection (d) scores for each group are also depicted in the remaining panels. Rain clouds represent the distribution of scores, individual points depict each participant's score, and the orange diamonds represent group means. Good Discriminators significantly outperformed the other two groups on all three measures, whereas Indiscriminate Believers and Indiscriminate Sceptics did not significantly differ from one another on any measure.

We compared these three groups on general knowledge, scientific reasoning, and cognitive reflection (see Figure 1). In each instance, the between‐groups one‐way ANOVA was significant. Follow up pairwise Tukey tests then revealed that, for each measure, Good Discriminators significantly outperformed the other two groups whereas Indiscriminate Believers and Indiscriminate Sceptics did not significantly differ from one another. Descriptive statistics and results are presented in Table 5.

**TABLE 5:** Descriptive statistics and group comparisons for each cognitive measure and all three grouping methods.

Variable		Grouping method									
		Thresholds			3‐profile LPA			4‐profile LPA			
		IB (*n* = 258) M_auc_ = .51 M_c_ = .77	IS (*n* = 205) M_auc_ = .56 M_c_ = −.77	GD (*n* = 200) M_auc_ = .80 M_c_ = −.37	IB (*n* = 258) M_auc_ = .48 M_c_ = .71	IS (*n* = 94) M_auc_ = .53 M_c_ = −1.25	GD (*n* = 311) M_auc_ = .75 M_c_ = −.31	IB (*n* = 91) M_auc_ = .44 M_c_ = 1.36	IS (*n* = 94) M_auc_ = .56 M_c_ = −1.35	GD (*n* = 241) M_auc_ = .77 M_c_ = −.40	AV (*n* = 237) M_auc_ = .54 M_c_ = .28
General knowledge	Mean (*SD*)	71.05 (12.75)	71.37 (13.54)	77.93 (12.81)	69.96 (12.78)	71.60 (13.07)	76.43 (13.21)	69.56 (13.25)	71.98 (13.45)	77.52 (12.98)	70.76 (12.66)
	Overall F (df) Pairwise comparisons	*F*(2, 660) = 18.76, *p* < .001 IB vs. IS: M_diff_ = 0.32, *p* = .961 IB vs. GD: M_diff_ = 6.88, *p* < .001 IS vs. GD: M_diff_ = 6.56, *p* < .001			*F*(2, 660) = 18.24, *p* < .001 IB vs. IS: M_diff_ = 1.64, *p* = .550 IB vs. GD: M_diff_ = 6.47, *p* < .001 IS vs. GD: M_diff_ = 4.83, *p* = .005			*F*(3, 659) = 14.38, *p* < .001 IB vs. IS: M_diff_ = 2.42, *p* = .584 IB vs. GD: M_diff_ = 7.96, *p* < .001 IS vs. GD: M_diff_ = 5.54, *p* = .003 IB vs. AV: M_diff_ = 1.20, *p* = .877 IS vs. AV: M_diff_ = 1.22, *p* = .867 GD vs. AV: M_diff_ = 6.76, *p* < .001			
Cognitive reflection	Mean (*SD*)	3.67 (1.94)	3.78 (2.02)	4.82 (1.80)	3.53 (1.95)	3.82 (2.08)	4.56 (1.88)	3.55 (2.06)	3.76 (2.05)	4.69 (1.84)	3.71 (1.93)
	Overall F (df) Pairwise comparisons	*F*(2, 660) = 22.95, *p* < .001 IB vs. IS: M_diff_ = 0.11, *p* = .815 IB vs. GD: M_diff_ = 1.15, *p* < .001 IS vs. GD: M_diff_ = 1.04, *p* < .001			*F*(2, 660) = 20.75, *p* < .001 IB vs. IS: M_diff_ = 0.29, *p* = .422 IB vs. GD: M_diff_ = 1.03, *p* < .001 IS vs. GD: M_diff_ = 0.74, *p* = .004			*F*(3, 659) = 13.90, *p* < .001 IB vs. IS: M_diff_ = 0.21, *p* = .888 IB vs. GD: M_diff_ = 1.14, *p* < .001 IS vs. GD: M_diff_ = 0.93, *p* = <.001 IB vs. AV: M_diff_ = 0.16, *p* = .903 IS vs. AV: M_diff_ = 0.04, *p* = .998 GD vs. AV: M_diff_ = 0.98, *p* < .001			
Scientific reasoning	Mean (*SD*)	5.63 (2.49)	6.17 (2.51)	7 .66 (2.54)	5.59 (2.39)	6.09 (2.50)	7.18 (2.68)	5.64 (2.53)	6.19 (2.53)	7.41 (2.61)	5.77 (2.47)
	Overall F (df) Pairwise comparisons	*F*(2, 660) = 38.16, *p* < .001 IB vs. IS: M_diff_ = 0.54, *p* = .055 IB vs. GD: M_diff_ = 2.03, *p* < .001 IS vs. GD: M_diff_ = 1.49, *p* < .001			*F*(2, 660) = 28.37, *p* < .001 IB vs. IS: M_diff_ = 0.49, *p* = .245 IB vs. GD: M_diff_ = 1.59, *p* < .001 IS vs. GD: M_diff_ = 1.10, *p* = .001			*F*(3, 659) = 20.48, *p* < .001 IB vs. IS: M_diff_ = 0.55, *p* = .448 IB vs. GD: M_diff_ = 1.77, *p* < .001 IS vs. GD: M_diff_ = 1.22, *p* = .001 IB vs. AV: M_diff_ = 0.13, *p* = .973 IS vs. AV: M_diff_ = 0.42, *p* = .529 GD vs. AV: M_diff_ = 1.64, *p* < .001			

Abbreviations: AV, Ambivalents; GD, Good Discriminators; IB, Indiscriminate Believers; IS, Indiscriminate Sceptics.

A similar pattern of results emerged when grouping participants via a Latent Profile Analysis (LPA) based on discriminability and response bias scores. When we forced three profiles, the groups were similar to those above. A completely data‐driven LPA, however, suggested four profiles were appropriate, with the fourth profile capturing participants with poor discriminability and middling response bias (i.e. ‘Ambivalents’). Once again, Good Discriminators outperformed the other three groups on each cognitive measure, whereas the other groups did not differ significantly from one another on any measure.

#### Preregistered analyses

Similar to Study 1, participants were pre‐screened several months prior using four implausible conspiracy items rated on a 0–100 scale (Martire et al., 2020; Martire et al., 2023). However, these items were re‐administered during the study itself, and participants were formally grouped as either Endorsers (rated any claim >60 out of 100) or Non‐endorsers (rated all four claims <40 out of 100) based on in‐study responses. In all, there were 332 Endorsers and 295 Non‐endorsers (*N* = 627), with 36 no longer falling into either group. A priori power analyses indicated that a sample of 600 provides 80% power to detect small‐to‐medium group differences (Cohen's *d* = 0.23).

Our first prediction was that, compared to Non‐endorsers, Endorsers would more strongly believe in both unverified and verified conspiracies (Cohen's *d*s > .5), reflecting that those who believe false conspiracies also tend to believe true ones. As predicted, Endorsers (*M* = 4.86, *SD* = 2.05) believed unverified conspiracies more than Non‐endorsers did (*M* = 2.22, *SD* = 1.35), *t*(625) = 18.82, *p* < .001, *d* = 1.51. They also believed verified conspiracies (*M* = 5.38, *SD* = 1.82) more than Non‐endorsers did (*M* = 4.19, *SD* = 1.75), *t*(625) = 8.37, *p* < .001, *d* = 0.67.

Second, we planned to compare these groups on discriminability (AUC) but we had no a priori predictions. Endorsers were poorer (*M* = 0.54, *SD* = 0.14) than Non‐endorsers (*M* = 0.70, *SD* = 0.14) at discriminating between verified and unverified conspiracies, *t*(625) = −14.94, *p* < .001, *d* = −1.20. However, we later realized that the grouping method introduces a selection bias because Non‐endorsers are both more likely to include those with a conservative response bias and those who discriminate effectively. This is a key reason why considering three groups is more appropriate.

Finally, we expected Endorsers to report higher conspiracy mentality and lower trust in experts than Non‐endorsers,^1^ and that these variables would correlate with response bias on the conspiracy items. As predicted, Endorsers had higher conspiracy mentality (*M* = 7.54, *SD* = 1.24) than Non‐endorsers (*M* = 5.72, *SD* = 1.60), *t*(625) = 16.01, *p* < .001, *d* = 1.28, and lower trust in experts (*M* = 3.51, *SD* = 1.36) than Non‐endorsers (*M* = 5.35, *SD* = 0.90), *t*(625) = −19.63, *p* < .001, *d* = −1.57. Table 3 also shows that response bias was moderately positively correlated with conspiracy mentality, and moderately negatively correlated with trust in experts. A conspiracist worldview and distrust of experts might therefore serve as heuristics people use to determine whether conspiracy claims are true.

## STUDY 3

To follow up the exploratory analyses from Study 2, we conducted a third, preregistered study. Our first aim was to re‐run bivariate correlations to confirm relationships between key variables. We expected (a) strong positive correlations between belief in unverified conspiracies, belief in verified conspiracies, and response bias, (b) moderate positive intercorrelations between our three measures of cognitive sophistication, (c) small negative associations between measures of cognitive sophistication and belief in unverified conspiracies, but non‐significant associations between these measures and belief in verified conspiracies, and (d) moderate positive associations between the cognitive sophistication measures and discrimination (AUC).

The second aim was to confirm which variables predict conspiracy discrimination by re‐running a multiple linear regression analysis. In line with Study 2, we expected general knowledge, scientific reasoning, trust in experts, conspiracy mentality, political conservatism, age and gender to uniquely predict discriminability. Additionally, for each cognitive measure, we compared two regression models—one with only response bias as a predictor, and one with both response bias and discrimination—to test whether discrimination is a stronger predictor than response bias. We expected response bias alone to negatively predict each measure, but only discrimination to be a significant (positive) predictor when both are included.

The final aim was to re‐examine our comparison of ‘Good discriminators’, ‘Indiscriminate Sceptics’ and ‘Indiscriminate Believers’ on cognitive sophistication. In line with Study 2, we expected Good Discriminators to outperform Indiscriminate Sceptics and Indiscriminate Believers on our three cognitive measures, but for Indiscriminate Sceptics and Indiscriminate Believers not to differ statistically from one another.

### Methods

The methods for Study 3 were similar to Study 2, with some minor differences. Participants were recruited in the same way, but pre‐screening took place directly before the study. We also recruited a gender‐balanced sample entirely from the United States. The general knowledge items were also converted to true‐false questions, with scores computed as percentage correct (Range: 0 to 1).

We preregistered to recruit up to 350 participants. This was based on available funding, but a priori power analyses also indicated that a sample of this size ensures sufficient statistical power (>80%) to detect small‐to‐medium effect sizes (*f* > .20) in one‐way ANOVAs with three groups. This effect size estimate is conservative given the main effects from Study 2. A sample of this size also provides 80% power to detect effects of *f* > .06 in multiple regression analyses involving up to 12 predictors.

Of the 350 participants recruited, seven were excluded for failing at least one attention check, in line with the preregistration. This left a final sample of 343 participants. The mean age was 46.0 (*SD* = 13.4) and the sample was split evenly between genders (51.0% woman/female; 48.4% man/male; 0.6% non‐binary). Most participants (69.1%) identified as White/Caucasian (11.4% African American; 9.3% Asian; 7.6% Hispanic; 2.3% other; 0.3% did not say). Most (72.6%) also had tertiary level education or more (27.1% secondary education or less; 0.3% did not say) and the vast majority (95.6%) reported English as their first language (4.4% said it was not).

### Results and discussion

#### Relationships between variables

Table 6 displays the zero‐order correlations for Study 3. Many of the relationships aligned with Study 2 and our preregistered predictions. There were strong positive correlations between belief in unverified conspiracies, belief in verified conspiracies and response bias. There were also moderate positive intercorrelations between our measures of cognitive sophistication (general knowledge, cognitive reflection and scientific reasoning). Additionally, we found small, negative correlations between belief in unverified conspiracies and the three cognitive sophistication measures, and non‐significant correlations between belief in verified conspiracies and both cognitive reflection and scientific reasoning. However, there was a small but significant positive correlation between belief in verified conspiracies and general knowledge. Finally, distinguishing between verified and unverified conspiracies (AUC) was moderately correlated with scores on the three cognitive sophistication measures. In addition to these preregistered predictions, AUC was positively correlated with trust in experts, age and education, and negatively correlated with conspiracy mentality and political conservatism.

**TABLE 6:** Descriptive statistics and zero‐order correlations between variables in Study 3.

	*M*	*SD*	1	2	3	4	5	6	7	8	9	10	11	12	13	14
1. Unverified consp. belief	3.61	2.30	–													
2. Verified consp. belief	5.21	2.00	0.51**	–												
3. Response bias (c)	0.05	0.84	0.86**	0.80**	–											
4. Discriminability (AUC)	0.63	0.17	−0.62**	0.29**	−0.24**	–										
5. General knowledge	0.74	0.15	−0.26**	0.18**	−0.07	0.41**	–									
6. Scientific reasoning	6.66	2.86	−0.34**	0.06	−0.17**	0.41**	0.40**	–								
7. Cognitive reflection	4.50	2.16	−0.29**	0.04	−0.18**	0.34**	0.43**	0.49**	–							
8. Conspiracy mentality	6.41	1.74	0.71**	0.43**	0.64**	−0.39**	−0.14*	−0.28**	−0.20**	–						
9. Trust in experts	4.43	1.50	−0.44**	−0.16**	−0.34**	0.34**	0.17**	0.15**	0.14**	−0.37**	–					
10. Conservatism	4.26	2.01	0.31**	0.15**	0.28**	−0.24**	−0.11*	−0.14**	−0.09	0.30**	−0.54**	–				
11. SES	5.07	1.83	−0.14**	−0.07	−0.11*	0.04	0.16**	0.13*	0.19**	−0.11*	0.12*	0.02	–			
12. Age	46.03	13.40	−0.14**	−0.02	−0.10	0.15**	−0.06	0.02	0.00	−0.18**	−0.18**	0.14*	−0.02	–		
13. Education	2.95	0.80	−0.21**	−0.08	−0.17**	0.15**	0.18**	0.21**	0.24**	−0.18**	0.14*	0.00	0.38**	0.09	–	
14. Gender	*–*	*–*	0.00	0.08	0.04	0.08	0.07	0.02	0.09	−0.07	0.08	0.05	−0.03	−0.16**	−0.01	–
15. Native English	*–*	*–*	0.12*	0.15**	0.15**	−0.01	−0.01	−0.03	−0.01	0.13*	−0.10	0.11*	−0.02	0.07	−0.08	−0.02

*Note*: For analyses of gender, 0 = woman/female, 1 = man/male. For analyses of English‐speaking status, 0 = non‐native speaker, 1 = native speaker.

* *p* < .05;

** *p* < .01.

#### Predicting discriminability

We used a linear regression analysis to reassess the unique predictors of conspiracy discriminability (see Table 7). The predictors together explained 38.9% of the variance. Consistent with Studies 1 and 2, and with preregistered hypotheses, general knowledge and scientific reasoning scores predicted better discrimination. Being older, less conspiracy minded and more trusting of experts also predicted better discrimination, as predicted. However, political conservatism and gender were not significant independent predictors.

**TABLE 7:** Predicting discrimination of verified and unverified conspiracies (AUC) in Study 3.

Predictor	*β*	*b*	*SE*	*t*‐value	*p*‐value
Intercept	–	0.2	.08	2.98	.003
General knowledge	.25	0.29	.06	4.96	<.001
Scientific reasoning	.20	0.01	.00	3.83	<.001
Cognitive reflection	.08	0.01	.00	1.61	.108
Age	.18	0.00	.00	3.83	<.001
Education	.00	0.00	.01	.010	.992
Gender	−.05	0.02	.01	−1.11	.269
Native English	−.04	−0.01	.02	−.080	.423
Conspiracy mentality	−.17	−0.02	.01	−3.39	.001
Trust in experts	.20	0.02	.01	3.68	<.001
Political conservatism	−.04	0.00	.00	−0.73	.467
SES	−.07	−0.01	.00	−1.44	.150

*Note*: For analyses of gender, 0 = woman/female, 1 = man/male. For analyses of English‐speaking status, 0 = non‐native speaker, 1 = native speaker.

#### Predicting cognitive sophistication

As preregistered, we flipped the analytic direction by conducting linear regression analyses to assess whether AUC more strongly predicts cognitive sophistication compared to response bias. When response bias was the only predictor, it did not significantly predict general knowledge (*b* = −0.01, *p* = .197), but it was a significant negative predictor for cognitive reflection (*b* = −0.47, *p* = .001) and scientific reasoning (*b* = −0.59, *p* = .001). When AUC was added as a second predictor, however, response bias was either no longer significant or a much weaker predictor (general knowledge: *b* = 0.01, *p* = .563; cognitive reflection: *b* = −0.28, *p* = .040; scientific reasoning: *b =* −0.27, *p* = .122). AUC, by contrast, was consistently a significant, positive predictor of cognitive sophistication (general knowledge: *b* = 0.36, *p* < .001; cognitive reflection: *b* = 3.96, *p* < .001; scientific reasoning: *b =* 6.53, *p* < .001).

#### Comparing correlations with conspiracy mentality

Though not preregistered, we conducted a dependent samples Fisher's r‐to‐z test to compare conspiracy mentality's association with response bias and discriminability. Conspiracy mentality was more strongly associated with response bias than with discriminability, *z* = 21.97, *p* < .001. This suggests that conspiracy mentality reflects response tendency rather than accurate discrimination.

#### Group comparisons

**FIGURE 2:**
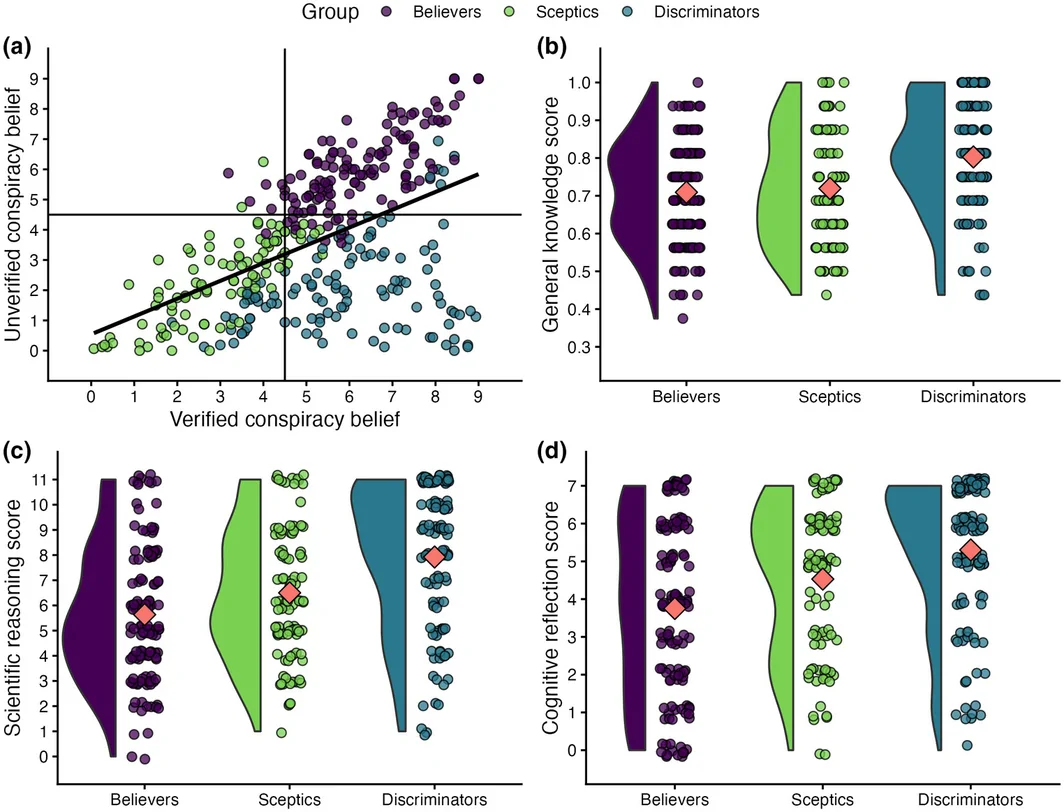
Comparison of three groups on cognitive ability measures in Study 3. In Panel a, Indiscriminate Believers (purple), Indiscriminate Sceptics (green), and Good Discriminators (dark blue) are plotted according to their mean belief in unverified and verified conspiracies. The regression line represents the correlation between the two. General knowledge (b), scientific reasoning (c), and cognitive reflection (d) scores for each group are depicted in the remaining panels. Rain clouds represent score distributions, individual points depict each participant's score and orange diamonds represent group means. Good Discriminators significantly outperformed the other two groups on all three measures. Indiscriminate Sceptics outperformed Indiscriminate Believers on scientific reasoning and cognitive reflection, but not general knowledge. When differences in discrimination were controlled for, however, the three groups did not significantly differ on any measure.

For our comparison of groups, the results mostly aligned with hypotheses, but there were some deviations (see Figure 2; Table 8). We grouped participants into ‘Good Discriminators’ (AUC > .7), ‘Indiscriminate Believers’ (AUC < .7; *c* > 0) and ‘Indiscriminate Sceptics’ (AUC < .7, *c* < 0) based on principled thresholds. The ANOVAs for each cognitive sophistication measure were significant. Follow‐up analyses showed that Good Discriminators, as anticipated, outperformed Indiscriminate Sceptics and Indiscriminate Believers on general knowledge, cognitive reflection and scientific reasoning. Indiscriminate Sceptics and Indiscriminate Believers did not differ statistically for general knowledge, as expected. However, Indiscriminate Sceptics outperformed Indiscriminate Believers on cognitive reflection and scientific reasoning.

**TABLE 8:** Descriptive statistics and group comparisons for each cognitive measure and both grouping methods in Study 3.

Variable		Grouping method					
		Thresholds			3‐profile LPA		
		IB (*n* = 129) M_auc_ = .50 M_c_ = .82	IS (*n* = 98) M_auc_ = .57 M_c_ = −.71	GD (*n* = 116) M_auc_ = .83 M_c_ = −.15	IB (*n* = 127) M_auc_ = .50 M_c_ = .86	IS (*n* = 118) M_auc_ = .60 M_c_ = −.71	GD (*n* = 98) M_auc_ = .84 M_c_ = −.06
General knowledge	Mean (*SD*)	0.71 (0.13)	0.72 (0.15)	0.80 (0.14)	0.71 (0.14)	0.72 (0.15)	0.82 (0.13)
	Overall F (df) Pairwise comparisons	*F*(2, 340) = 15.20, *p* < .001 IB vs. IS: M_diff_ = 0.01, *p* = .857 IB vs. GD: M_diff_ = 0.09, *p* < .001 IS vs. GD: M_diff_ = 0.08, *p* < .001			*F*(2, 340) = 22.97, *p* < .001 IB vs. IS: M_diff_ = 0.01, *p* = .728 IB vs. GD: M_diff_ = 0.12 *p* < .001 IS vs. GD: M_diff_ = 0.11, *p* < .001		
Cognitive reflection	Mean (*SD*)	3.75 (2.29)	4.53 (1.98)	5.30 (1.85)	3.76 (2.37)	4.52 (1.95)	5.44 (1.72)
	Overall F (df) Pairwise comparisons	*F*(2, 340) = 17.23, *p* < .001 IB vs. IS: M_diff_ = 0.78, *p* = .014 IB vs. GD: M_diff_ = 1.55, *p* < .001 IS vs. GD: M_diff_ = 0.77, *p* = .019			*F*(2, 340) = 18.51, *p* < .001 IB vs. IS: M_diff_ = 0.76, *p* = .011 IB vs. GD: M_diff_ = 1.68, *p* < .001 IS vs. GD: M_diff_ = 0.92, *p* = .003		
Scientific reasoning	Mean (*SD*)	5.64 (2.65)	6.50 (2.60)	7.92 (2.83)	5.63 (2.69)	6.59 (2.55)	8.06 (2.86)
	Overall F (df) Pairwise comparisons	*F*(2, 340) = 22.17, *p* < .001 IB vs. IS: M_diff_ = 0.86, *p* = .046 IB vs. GD: M_diff_ = 2.29, *p* < .001 IS vs. GD: M_diff_ = 1.42, *p* < .001			*F*(2, 340) = 22.56, *p* < .001 IB vs. IS: M_diff_ = 0.96, *p* = .015 IB vs. GD: M_diff_ = 2.43, *p* < .001 IS vs. GD: M_diff_ = 1.47, *p* < .001		

Abbreviations: GD, Good Discriminators; IB, Indiscriminate Believers; IS, Indiscriminate Sceptics.

We also grouped participants using a LPA based on the same clustering models as in Study 2, and this suggested three profiles. Results for the profile‐based group comparisons were similar to the threshold‐based comparisons. All three ANOVAs were significant. Pairwise comparisons revealed that Good Discriminators outperformed the other two groups on each measure. Indiscriminate Sceptics did not differ from the Indiscriminate Believers on general knowledge, but they outperformed Indiscriminate Believers on cognitive reflection and scientific reasoning.

That said, Indiscriminate Sceptics had a slightly higher mean AUC than Indiscriminate Believers under both grouping methods (see Table 8). Group differences in discriminability could therefore explain why Indiscriminate Sceptics scored modestly higher than Indiscriminate Believers on some cognitive measures. To test this, we followed each ANOVA above with an ANCOVA controlling for AUC. If the observed group differences are driven by discrimination rather than response bias, group differences should disappear once AUC is accounted for. Indeed, none of the groups differed significantly across all pairwise comparisons based on these ANCOVAs (*p*s > .05).

These findings, together with the linear regression analyses from earlier, demonstrate that conspiracy discrimination is a stronger predictor of cognitive sophistication than response bias. This supports the central claim from Study 2 that cognitive sophistication is more closely tied to accurately distinguishing verified from unverified conspiracies than a tendency to either believe or disbelieve conspiracies.

## GENERAL DISCUSSION

Although some conspiracy claims lack empirical support (unverified), others are on the official public record (verified). Yet, research has focused almost exclusively on belief in the former. Across three studies, we examined what predicts the capacity to distinguish between verified and unverified conspiracies, whether discrimination is a stronger predictor of cognitive sophistication than response bias, and whether indiscriminate rejection, like indiscriminate belief, is associated with lower cognitive sophistication. We found that better discriminators consistently outperform poorer discriminators on tests of general knowledge, scientific reasoning and cognitive reflection. When combined in a regression model with other predictors, general knowledge and scientific reasoning ability also remained as significant predictors. Additionally, when flipping the analytic direction, discriminability was consistently a stronger predictor of cognitive sophistication than response bias.

In Studies 2 and 3, we also classified participants into three groups: Good Discriminators, Indiscriminate Believers and Indiscriminate Sceptics. Good Discriminators outperformed the other two groups on each cognitive sophistication measure, whereas Indiscriminate Believers and Indiscriminate Sceptics did not differ significantly on any measure in Study 2. This was also the case in Study 3 once differences in discriminability were controlled for.

Prior research consistently links belief in unverified or implausible conspiracies to poorer domain‐general reasoning (Binnendyk & Pennycook, 2022; Gjoneska, 2021; Lantian et al., 2021; Pytlik et al., 2020; Yelbuz et al., 2022), implicitly reinforcing the idea that rejecting conspiracies must signal cognitive sophistication. However, conspiracy beliefs are commonly defined and measured using only unwarranted or implausible conspiracy theories. By examining belief in both verified and unverified conspiracy claims, we show that blindly dismissing conspiracy claims also appears to be associated with lower cognitive performance. In other words, effective discrimination between verified and unverified conspiracies, rather than indiscriminate belief or rejection, is a better indicator of cognitive sophistication.

This problem may run deeper still. In Studies 2 and 3, conspiracy mentality—a widely used predictor of conspiracy belief—was associated with both a liberal response bias and reduced discrimination, but its association with response bias was significantly stronger. Research relying on conspiracy mentality scales may therefore primarily capture a tendency to indiscriminately endorse conspiracy claims rather than a deficit in discrimination, further compounding the limitations of existing research in this area (see Tagand et al., 2026). That said, response bias showed small negative associations with cognitive sophistication measures in all three studies, such that those who tend to reject conspiracy claims were somewhat more cognitively sophisticated than those who tend to endorse them. This speaks against a notion of strict symmetry between sceptics and believers (see Tagand et al., 2026). The LPAs nevertheless suggest that Indiscriminate Sceptics represent a genuine participant profile that emerges naturally from the data. Crucially, this group displayed comparable cognitive sophistication to Indiscriminate Believers in Study 2, and their advantage in Study 3 disappeared when differences in discriminability were controlled for.

Prior work has indicated that poorer reasoning predicts belief in implausible conspiracies but not plausible ones (Frenken et al., 2024; Hattersley et al., 2022). We conceptually replicate this work, demonstrating that scientific reasoning and cognitive reflection are negatively related to belief in unverified conspiracies but unrelated to belief in verified conspiracies. However, general knowledge was sometimes negatively correlated with unverified conspiracy belief and positively correlated with verified conspiracy belief, suggesting that historical knowledge might help people recognize genuine conspiracies. Both types of belief were also positively correlated with conspiracy mentality and political conservatism and negatively correlated with trust in experts. This evidence suggests that processes driving belief in implausible/unverified conspiracies may differ from processes underpinning belief in plausible/verified ones (Lewandowsky et al., 2018; Stanovich & Toplak, 2025), but also that there is overlap.

We also extend this literature by focusing primarily on conspiracy discrimination, which considers belief in verified claims *relative to* unverified claims. Consistent with prior work (Bensley et al., 2020; Moulding et al., 2016; Pennycook et al., 2025; but see Wagner‐Egger et al., 2023), we found that verified and unverified conspiracy belief is strongly correlated, highlighting a potential trade‐off between incorrectly believing conspiracies that are false and incorrectly dismissing conspiracies that are true. Using an SDT approach, however, we could separate general credulity (response bias) from discriminability, revealing that discrimination is more closely tied to cognitive sophistication than response bias alone. Additionally, better discrimination correlated with political liberalism, higher trust in experts and lower conspiracy mentality in all three studies. Stronger discriminators also skewed male in Studies 1 and 2, and age positively predicted discrimination in Studies 2 and 3. Further research is nevertheless needed to better understand the correlates of conspiracy discrimination (see Stanovich & Toplak, 2025).

There are two caveats to our SDT approach. First, the ground truth of conspiracy claims is not directly available, and what researchers classify as true or false can reflect subjective epistemological assumptions (Uscinski et al., 2024); some unverified claims may in fact be true and some genuine conspiracies may never come to light given their secretive nature. Claims nevertheless vary in their level of evidential support, and using verified/unverified as a proxy for true/false allows for meaningful examination of how well people differentiate these claims. Second, unlike typical discrimination tasks, participants were not explicitly asked to categorize claims as verified or unverified. Our measure of discrimination is therefore indirect, reflecting beliefs formed over a lifetime of exposure to evidence.

Finally, none of this implies that all conspiracy claims are true or that conspiracy theorists expose genuine conspiracies. Conspiracies are typically uncovered by professional investigators who collect evidence like confessions and official documents (Wagner‐Egger et al., 2019, 2023). When evaluating conspiracy claims that one cannot personally investigate, however, calibrated skepticism—differentiating claims based on evidential support rather than indiscriminate belief or indiscriminate rejection—appears most appropriate.

## CONCLUSIONS

There has been significant focus on the flawed cognition associated with believing unverified conspiracies. Here we find that a tendency to reject conspiracy claims is somewhat related to cognitive sophistication, but our central conclusion is that indiscriminate disbelief, like indiscriminate belief in conspiracies, is also a marker of poor cognitive sophistication. We therefore suggest that researchers consider measuring belief in both verified (plausible) and unverified (implausible) claims, and use signal detection approaches that have already been adopted in other areas of misinformation research (e.g. fake news; Batailler et al., 2022). This might offer a valuable way to examine trade‐offs in conspiracy thinking. For example, evaluating interventions using SDT can clarify whether they lower credulity or improve conspiracy discrimination (see O'Mahony et al., 2024). Considering both response bias and discrimination could offer a richer understanding of the causes and consequences of conspiracy belief.

## AUTHOR CONTRIBUTIONS

**Samuel G. Robson:** Conceptualization; formal analysis; investigation; methodology; project administration; resources; software; validation; visualization; writing – original draft; writing – review and editing. **Kristy A. Martire:** Conceptualization; funding acquisition; methodology; software; supervision; writing – review and editing. **Samuel Pearson:** Conceptualization; formal analysis; methodology; validation; writing – review and editing. **Tylor J. Cosgrove:** Conceptualization; methodology; software; validation; writing – review and editing. **Jolanda Jetten:** Funding acquisition; writing – review and editing; conceptualization. **Kate Faasse:** Conceptualization; funding acquisition; supervision. **Matthew J. Hornsey:** Conceptualization; funding acquisition; methodology; software; supervision; writing – review and editing.

## CONFLICT OF INTEREST STATEMENT

The authors declare no conflicts of interest.

## Data Availability

Materials, data and analytic scripts for Studies 1 and 2 can be found via Study 2's OSF project: https://osf.io/pnavw and they can be found here for Study 3: https://osf.io/xsjr2.

## Footnotes

^1^
We mistakenly preregistered that Endorsers would have higher trust, but we always anticipated that they would score lower given our preregistered rationale that ‘extreme trust in authority…may mean dismissing or overlooking nefarious actions committed by authorities.’
